# Development and validation of a duplex real-time PCR assay for the diagnosis of equine piroplasmosis

**DOI:** 10.1186/s13071-018-2751-6

**Published:** 2018-03-02

**Authors:** Vladislav A. Lobanov, Maristela Peckle, Carlos L. Massard, W. Brad Scandrett, Alvin A. Gajadhar

**Affiliations:** 1 Centre for Food-borne and Animal Parasitology, Canadian Food Inspection Agency, Saskatoon, Saskatchewan Canada; 2 Department of Animal Parasitology, Veterinary Institute, Federal Rural University of Rio de Janeiro, Seropedica, Rio de Janeiro, Brazil

**Keywords:** Equine piroplasmosis, *Theileria equi*, *Babesia caballi*, Real-time PCR, Confirmatory assay

## Abstract

**Background:**

Equine piroplasmosis (EP) is an economically significant infection of horses and other equine species caused by the tick-borne protozoa *Theileria equi* and *Babesia caballi*. The long-term carrier state in infected animals makes importation of such subclinical cases a major risk factor for the introduction of EP into non-enzootic areas. Regulatory testing for EP relies on screening of equines by serological methods. The definitive diagnosis of EP infection in individual animals will benefit from the availability of sensitive direct detection methods, for example, when used as confirmatory assays for non-negative serological test results. The objectives of this study were to develop a real-time quantitative polymerase chain reaction (qPCR) assay for simultaneous detection of both agents of EP, perform comprehensive evaluation of its performance and assess the assay’s utility for regulatory testing.

**Results:**

We developed a duplex qPCR targeting the *ema-1* gene of *T. equi* and the *18S rRNA* gene of *B. caballi* and demonstrated that the assay has high analytical sensitivities for both piroplasm species. Validation of the duplex qPCR on samples from 362 competitive enzyme-linked immunosorbent assay (cELISA)-negative horses from Canada and the United States yielded no false-positive reactions. The assay’s performance was further evaluated using samples collected from 430 horses of unknown EP status from a highly endemic area in Brazil. This set of samples was also tested by a single-target 18S rRNA qPCR for *T. equi* developed at the OIE reference laboratory for EP in Japan, and a previously published single-target 18S rRNA qPCR for *B. caballi* whose oligonucleotides we adopted for use in the duplex qPCR. Matching serum samples were tested for antibodies to these parasites using cELISA. By the duplex qPCR, *T. equi*-specific 18S rRNA qPCR and cELISA, infections with *T. equi* were detected in 87.9% (95% confidence interval, CI: 84.5–90.7%), 90.5% (95% CI: 87.3–92.3%) and 87.4% (95% CI: 84.0–90.2%) of the horses, respectively. The *B. caballi* prevalence estimates were 9.3% (95% CI: 6.9–12.4%) by the duplex qPCR and 7.9% (95% CI: 5.7–10.9%) by the respective single-target qPCR assay. These values were markedly lower compared to the seroprevalence of 58.6% (95% CI: 53.9–63.2%) obtained by *B. caballi*-specific cELISA. The relative diagnostic sensitivity of the duplex qPCR for *T. equi* was 95.5%, as 359 of the 376 horses with exposure to *T. equi* confirmed by cELISA had parasitemia levels above the detection limit of the molecular assay. In contrast, only 39 (15.5%) of the 252 horses with detectable *B. caballi*-specific antibodies were positive for this piroplasm species by the duplex qPCR.

**Conclusions:**

The duplex qPCR described here performed comparably to the existing single-target qPCR assays for *T. equi* and *B. caballi* and will be more cost-effective in terms of results turnaround time and reagent costs when both pathogens are being targeted for disease control and epidemiological investigations. These validation data also support the reliability of the *ema-1* gene-specific oligonucleotides developed in this study for confirmatory testing of non-negative serological test results for *T. equi* by qPCR. However, the *B. caballi*-specific qPCR cannot be similarly recommended as a confirmatory assay for routine regulatory testing due to the low level of agreement with serological test results demonstrated in this study. Further studies are needed to determine the transmission risk posed by PCR-negative equines with detectable antibodies to *B. caballi*.

## Background

Equine piroplasmosis (EP) is a haemoprotozoan infection of horses and other members of the family Equidae caused by two intracellular apicomplexan parasites, *Theileria equi* [1] and *Babesia caballi* [2]. Infections with either or both of these parasites may cause severe acute disease characterized by fever, hemolytic anemia, hemoglobinuria, icterus, splenomegaly and occasionally death. Animals that recover from a primary infection remain chronically infected and become inapparent carriers with fluctuating levels of parasitemia. The carrier state in equines infected with *T. equi* lasts a lifetime. Infections with *B. caballi* may persist for years; however, there is a contention that infected equines are capable of clearing *B. caballi* without babesiacidal treatment. Persistently infected carriers serve as a reservoir for iatrogenic and biological transmission of these parasites [3, 4]. There is also evidence of transplacental transmission of *T. equi* from infected carrier mares to their fetuses that may lead to abortion, stillbirth or neonatal piroplasmosis [3, 5]. Vector-borne transmission of EP is mediated by ixodid tick species belonging to the genera *Dermacentor*, *Hyalomma* and *Rhipicephalus* [6]. However, a member of the genus *Amblyomma* has also been shown to be a competent intrastadial vector of *T. equi* associated with a recent incursion of this pathogen in the USA [7, 8].

Importation of carrier animals with no overt signs of disease is a major risk factor for the introduction of EP into non-enzootic areas. Countries that wish to achieve or maintain EP-free status must enact regulations to restrict the entrance of infected equines. Therefore, EP presents a significant impediment to international movement of horses for trade and for participation in international equestrian events [6, 9]. Sensitive and specific laboratory diagnostic methods are essential for revealing asymptomatic equines carrying these parasites. Blood smear microscopy provides a means of identifying the organisms during the acute stage of infection, but parasitemia levels are generally too low in the persistent stage of infection for reliable detection by this method [10, 11]. Definitive diagnosis of EP is usually accomplished using one or a combination of the following serological methods: indirect immunofluorescent antibody test (IFAT), cELISA and immunoblot assay. There is evidence of significantly higher sensitivity of IFAT compared to cELISA in detecting early infections with *T. equi* [12]. The complement fixation test (CFT) has high specificity, but lacks sensitivity in the chronic stage of infection [13, 14].

The cELISA utilizing the recombinant equi merozoite antigen 1 (EMA-1) protein of *T. equi* and a monoclonal antibody specific to this immunodominant surface protein is currently considered to be one of the most robust and sensitive methods for detecting antibodies to this organism in chronically infected animals [13, 15]. The assay has been validated using a number of different geographical isolates of *T. equi* [13, 16]. The currently available cELISA for *B. caballi* is based on specific inhibition of binding of a monoclonal antibody to the recombinant rhoptry-associated protein 1 (RAP-1) antigen of this parasite by serum antibodies of infected equines [17]. Although this assay generally has a higher diagnostic sensitivity than that of the CFT [17], it was unable to detect infections with certain geographical isolates of *B. caballi* due presumably to sequence heterogeneity in the *rap-1* gene and its product [18, 19]. The validity of the RAP-1 cELISA for use as a singular diagnostic test for *B. caballi* has thus been questioned [18, 20].

Recent advances in molecular diagnostics have led to the development of an array of sensitive detection methods for EP based on targeted amplification of specific genomic loci of *T. equi* and *B. caballi*. Among these methods are conventional PCR [10, 21, 22], ‘nested’ PCR (nPCR) [11, 23, 24], PCR followed by detection of amplicons by reverse line blotting with non-radioactive probes [25], loop-mediated isothermal amplification [26] and qPCR [27–30]. The highest analytical sensitivity values were reported for nPCR and qPCR methods. Thus, nPCR targeting the *ema-1* gene of *T. equi* has been shown to produce a positive result at a parasitemia level of 6 × 10^-6^% infected cells [11], whereas DNA of *B. caballi* was detected by a *rap-1* gene-specific nPCR in all serial dilutions of infected equine erythrocytes down to a single parasitized cell [24]. In addition to also being very sensitive, the advantages of qPCR over nPCR and other currently available PCR-based methods include speed, ease of performance, quantitative ability and a lower risk of carry-over contamination due to the absence of a need for post-amplification manipulation of the products [31]. Genomic sites targeted by described qPCR assays for EP include the *18S rRNA* gene of *T. equi* [30] and its homolog in *B. caballi* [27], as well as genes encoding immunodominant surface proteins of these parasites, such as the *ema-1* gene of *T. equi* [28, 32] and the 48 kDa merozoite rhoptry protein (*bc48*) gene of *B. caballi* [29]. There is evidence suggesting that the translation products of *bc48* and *rap-1* genes represent the same protein [17]. A duplex real-time PCR for simultaneous detection of both *T. equi* and *B. caballi* has also been described [29]. This assay utilized *ema-1* gene-specific primers and probe that were adopted from earlier studies where these oligonucleotides were used for the quantification of parasitemia levels of *T. equi* in experimentally infected animals by single-target qPCR [32, 33]. However, a suboptimal diagnostic sensitivity for that single-target qPCR assay was later reported when it was used to test field samples in South Africa [28], due to the *ema-1* gene sequence heterogeneity in a subset of isolates originating from that geographical region.

Effective protocols for chemotherapeutic clearance of persistent infections using a carbanilide derivative, imidocarb dipropionate, have been developed for both *T. equi* and *B. caballi* [12, 34, 35]. Serial negative PCR test results will likely remain one of the requirements for verifying successful parasite elimination post-treatment, broadening the applicability of well-validated PCR assays for the regulatory control of EP.

The objectives of this study were to adopt the already well-characterized *18S rRNA* gene-specific oligonucleotides for *B. caballi* developed by Bhoora et al. [27] and develop new compatible *T. equi*-specific primers and hydrolysis probe for incorporation into a new duplex qPCR assay, determine analytical sensitivities of this assay for both piroplasm species, and compare diagnostic performance of the duplex qPCR against that of the cELISA and selected single-target qPCR assays using known negative (diagnostic specificity) and unknown status field samples from an enzootic area (relative diagnostic sensitivity).

## Methods

### Field samples

#### Samples from horses located in non-enzootic areas for EP (*n* = 362)

Matching samples of serum and whole EDTA blood were obtained from an abattoir located in Canada in April 2012 from 30 Canadian horses and 30 horses imported from the USA. Additional archived cryopreserved matching samples of whole EDTA blood and serum collected from 302 horses and ponies in the USA during the period from 1987 to 2009 were obtained from the Gluck Equine Research Center (University of Kentucky, Lexington, KY, USA).

#### Samples from horses located in an enzootic area for EP (*n* = 430)

Matching samples of serum and EDTA blood were collected from 430 horses of unknown EP status (i.e. these animals had no apparent clinical symptoms of EP) residing in the state of Rio de Janeiro, Brazil in October 2013 to January 2014. All samples of whole blood and serum were stored until use at -80 °C and -20 °C, respectively.

### Optimization of the DNA extraction procedure

Total DNA was extracted from 200 μl of whole EDTA blood using the DNeasy Blood and Tissue Kit (Qiagen, Hilden, Germany). The manufacturer’s protocol was preliminarily evaluated for its efficacy in removing PCR inhibitors present in blood from DNA preparations. To that end, DNA was extracted on two occasions from a stabilate of blood from a *T. equi-*infected horse obtained from the National Veterinary Services Laboratories, United States Department of Agriculture (NVSL/USDA; Ames, IA, USA). The extractions were performed either in strict accordance with the manufacturer’s protocol, or with modifications that entailed two consecutive washes of the spin column containing DNA adsorbed to the filter with either Buffer AW1 or Buffer AW2, or the lysis of erythrocytes using RBC Lysis Solution (Qiagen, Germantown, MD, USA) followed by centrifugation and decanting of most of the supernatant containing PCR inhibitors released from the cells prior to the column purification. These DNA preparations were then analysed by duplex qPCR as described below, except that the amplifications were performed using the TaqMan Universal Master Mix II (Life Technologies, Warrington, UK) either with or without addition of 200 ng/μl of bovine serum albumin (BSA; non-acetylated 20 mg/ml solution in water; Sigma-Aldrich, St. Louis, MO, USA). Modifications that produced markedly reduced and more consistent quantification cycle (Cq) values in the duplex qPCR were incorporated into the final protocol.

The final modified DNA extraction procedure was as follows. Blood samples were transferred into tubes pre-filled with 600 μl of RBC Lysis Solution (Qiagen) and mixed by inverting each tube a few times. This was followed by 5 min incubation at room temperature, a brief vortexing, and continued incubation for 5 min. After a brief vortexing, the tubes were centrifuged at 17,000× *g* for 30 min at 4 °C, and then most of the supernatant was discarded without disturbing the pellet. The remaining volume was adjusted to approximately 200 μl with nuclease-free water (Life Technologies, Grand Island, NY, USA). The pellet was thoroughly re-suspended and processed according to the kit’s protocol, except that two consecutive washes of the spin column with Buffer AW1 were performed.

### Development of the duplex real-time PCR

Twenty-two nucleotide sequences of the *ema-1* gene of *T. equi*, namely L13784, AB015208-AB015220, AB015235, AB043618, AF255730, AF261824, AY058899, DQ250541, U97167 and U97168, were retrieved from GenBank and aligned using BioEdit 7.0.9 [36]. Primers and a minor groove binder (MGB) hydrolysis probe were designed using the AlleleID 7.72 software (Premier Biosoft, Palo Alto, CA, USA). The software parameters were set to avoid cross-homology with the equine genome and cross-hybridization interference with the adopted oligonucleotides specific to the *18S rRNA* gene of *B. caballi* [27]. The PCR product amplified in the duplex qPCR using the *ema-1* gene-specific primers represents a 98 base pair fragment containing 89 nucleotides of the 3' extremity of this gene. Primers used in this study were synthesized by Integrated DNA Technologies (Coralville, IA, USA), whereas TaqMan MGB probes were obtained from Life Technologies (Carlsbad, CA, USA) (Table 1).

**Table 1:** Nucleotide sequences of primers and TaqMan MGB probes used in the duplex qPCR assay

Oligonucleotide name	Nucleotide sequence (5'-3') and modifications	Reference
TeEMA1-F	CTGACTACAAGGTYGTATAC	This study
TeEMA1-R	TGTCGTCACTTAGTAAAATAGA	This study
TeEMA1-P	*6*-*FAM*-TTCTCCGTCTATGGCGCA-*MGB*-*NFQ*	This study
Bc_18SF402	GTAATTGGAATGATGGCGACTTAA	Bhoora et al. [27]
Bc_18SR496	CGCTATTGGAGCTGGAATTACC	Bhoora et al. [27]
Bc_18SP	*VIC*-CCTCGCCAGAGTAA-*MGB*-*NFQ*	Bhoora et al. [27]

*Abbreviations*: Y, T or C; *MGB*, minor groove binder; *NFQ*, non-fluorescent quencher

Real-time qPCR reactions were performed in a CFX96 Real-Time PCR Detection System (Bio-Rad, Hercules, CA, USA). Each 25 μl duplex qPCR reaction contained 400 nM of each TeEMA1-F and TeEMA1-R primer, 480 nM of each Bc_18SF402 and Bc_18SR496 primer, 200 nM of each TeEMA1-P and Bc_18SP probe and 2 μl of DNA preparation in 1× TaqMan Environmental Master Mix 2.0 (Life Technologies). Supplementing the duplex qPCR reaction mixture with BSA was unnecessary, as the fully optimized assay was performed using a TaqMan PCR master mix with proprietary formulation designed to reduce the effect of common PCR inhibitors. The optimized cycling protocol consisted of the initial activation cycle at 95 °C for 10 min, followed by 45 cycles of 30 s at 95 °C, 30 s at 57 °C and 20 s at 72 °C with fluorescence data acquisition after the elongation step. Negative controls consisting of nuclease-free water and DNA extracted from blood of an EP-free horse (Western College of Veterinary Medicine (WCVM), University of Saskatchewan, Saskatoon, Canada) were included on each plate. Preparations of DNA extracted from stabilates of blood from horses infected with either *T. equi* or *B. caballi* (NVSL/USDA) were used as positive controls. Test samples with Cq values lower than 40 were considered positive.

To produce a plasmid containing the full-length *ema-1* gene of *T. equi*, DNA extracted from a stabilate of blood infected with a Peru strain of *T. equi* (NVSL/USDA) was amplified by PCR using primers (TeEMA1-Start) 5'-ATG ATT TCC AAA TCC TTT GC-3' and TeEMA1-R (Table 1). The amplification product was cloned into the pCR4-TOPO vector (Life Technologies), and the resulting plasmid was named pCR4-ema-1. The accuracy of cloning was confirmed by Sanger nucleotide sequencing that was performed at the National Research Council, Plant Biotechnology Institute (Saskatoon, Canada).

### Evaluation of the duplex real-time PCR performance

Amplification efficiency (E) values were calculated by the CFX Manager software (Bio-Rad) from slopes of standard curves produced by amplification of serially diluted templates (standards) according to the equation E = (10^-(1/Slope)^ – 1) × 100%. The linearity of data (*R*^2^ > 0.98), an E value within the range of 90–110% and consistency of Cq values across replicates were considered indicative of a well-optimized qPCR.

A fresh sample of *T. equi*-infected equine blood suitable for determining the level of parasitemia by microscopy was not available for this study. Therefore, the analytical sensitivity of the duplex qPCR assay for detecting *T. equi* was evaluated using standards consisting of ten-fold serial dilutions of the plasmid pCR4-ema-1 linearized with the restriction enzyme *Sca*I. The digestion product was purified from agarose gel using the QIAquick Gel Extraction kit (Qiagen) and its DNA concentration was measured using the NanoDrop 2000c spectrophotometer (Thermo Scientific, Wilmington, DE, USA). Dilutions of the fragment with specified numbers of *ema-1* copies were prepared according to guidelines provided in the Applied Biosystems tutorial [37]. Four replicates of each dilution of these standards were amplified in the presence or absence of background DNA represented by 250 ng per reaction of DNA from blood of an EP-free horse.

To determine the analytical sensitivity of the duplex qPCR for *B. caballi*, duplicate ten-fold serial dilutions of a sample of equine blood with 0.4% parasitemia (NVSL/USDA) were prepared in uninfected equine blood. The level of parasitemia was determined by microscopy performed at 1,000× magnification on thin blood smears stained using a Diff-Quik Stain Set (Newark, DE, USA), and counting the proportion of infected erythrocytes in half of each of five fields of view. Total DNA was extracted from these dilutions according to the protocol described above. In addition, DNA extracted from an undiluted sample of this infected blood was used to prepare ten-fold serial dilutions in TE buffer (10 mM Tris-HCl pH 8.0, 1 mM EDTA). These two differently prepared sets of standards were amplified in quadruplicate by the duplex qPCR.

The diagnostic specificity of the duplex qPCR assay was evaluated by testing matching samples of whole blood and serum from the negative horse population by this molecular assay and cELISA, respectively. The relative diagnostic sensitivity and specificity of the duplex qPCR were assessed using samples collected from 430 horses of unknown infection status from an enzootic area for EP in Brazil. The sera were tested for antibodies to both *T. equi* and *B. caballi* by cELISA. The samples of whole blood were tested by the duplex qPCR and an established qPCR assay targeting the *18S rRNA* gene of *T. equi* [30]. Moreover, to further confirm whether the performance of the adopted primers and probe for *B. caballi* in the duplex qPCR would be as robust as that under the original conditions, we tested these samples by the single-target *B. caballi* 18S rRNA qPCR according to the published protocol [27].

### Competitive ELISA

All samples of serum were tested for the presence of antibodies to *B. caballi* and *T. equi* by cELISA using kits manufactured by VMRD (Pullman, WA, USA). The assays were performed in strict accordance with the manufacturer’s protocols, whereby a test sample producing ≥ 40% inhibition was considered positive. Optical density values were obtained using a SpectraMax Plus 384 microplate reader (Molecular Devices, Sunnyvale, CA, USA).

### Statistical analysis of the data

To compare amplification of different sets of standards in the duplex qPCR, standard curves were generated using Prism 6 software (GraphPad Software, La Jolla, CA, USA) by plotting Cq values against template dilutions or gene copies by non-linear regression (Semilog line). The slope values of these standard curves were equal to those generated by the CFX Manager software (Bio-Rad) for the same qPCR datasets. The slopes of standard curves were then compared using the extra sum-of-squares *F*-test (Prism 6). The data were considered significantly different if the *P*-value was less than 0.05.

Calculations of Kappa [38], the 95% CI for a proportion (by the modified Wald method [39]), and positive and negative predictive values of the duplex qPCR were performed using QuickCalcs online resources (Categorical data; GraphPad Software). The 95% CI values for replicates were calculated using Prism 6. For the Kappa statistic, the software assigns descriptors to the Kappa coefficient (*κ*) value ranges based on the scheme from Altman [38], where *κ* < 2 = poor agreement; 0.21–0.4 = fair agreement; 0.41–0.6 = moderate agreement; 0.61–0.8 = good agreement; 0.81–0.99 = very good agreement.

## Results

### Optimization of the DNA extraction procedure

**Fig. 1:**
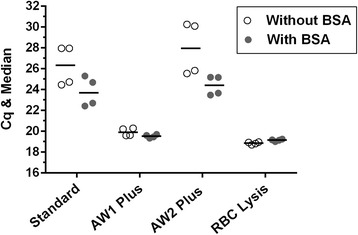
Optimization of the DNA extraction procedure. Individual Cq values with median after the duplex qPCR amplification of DNA extracted from *T. equi*-infected equine blood according to the standard protocol provided with the DNA extraction kit or with modifications that involved preliminary lysis of erythrocytes with RBC Lysis Solution or additional wash of the spin column containing adsorbed DNA with either Buffer AW1 (AW1 Plus) or Buffer AW2 (AW2 Plus). These samples were amplified either with or without BSA in the reaction mixture. Graph was produced using Prism 6

At an early stage of the duplex qPCR development, it was noted that the amplification of DNA preparations extracted strictly according to the spin column-based manufacturer’s protocol from duplicate aliquots of a sample of equine blood infected with either of the two piroplasm species produced Cq values that varied widely between preparations. However, markedly lower and more consistent Cq values were obtained when ten-fold dilutions of these same DNA preparations were amplified. This indicated the presence of varying amounts of PCR inhibitors in template preparations extracted according to the kit’s protocol. To address this problem, we assessed individual effects of several modifications to the DNA extraction procedure on Cq values in the duplex qPCR. A noticeable improvement in PCR amplification upon addition of BSA to the reaction mixture implies the presence of inhibitory substances in the template preparation [40]. Reduced inhibition in the presence of BSA was pronounced when DNA templates prepared according to the manufacturer’s protocol, or to the protocol modified by introducing one additional wash of the spin column containing adsorbed DNA with Buffer AW2, were amplified (Fig. 1). However, even without BSA, modifications entailing the lysis of erythrocytes prior to DNA extraction or an additional wash of the spin column with Buffer AW1 resulted in markedly lower and more consistent Cq values. Therefore, both these modifications were incorporated into the DNA extraction protocol used in this study (see Methods).

### Amplification efficiency and analytical sensitivity of the duplex qPCR

**Fig. 2:**
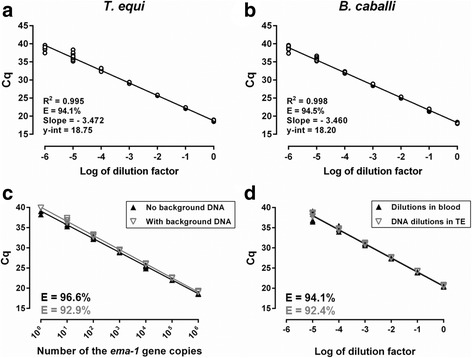
Standard curves for *T. equi* (**a**) and *B. caballi* (**b**) generated by plotting pooled Cq values from 5 separate amplifications of standards representing a 6-log dynamic range of starting template quantity (serially diluted DNA extracted from stabilates of infected equine blood) against the log of template dilution factor. Determination of the analytical sensitivity of the duplex qPCR assay for *T. equi* (**c**) and *B. caballi* (**d**). In **c**, standard curves were produced by amplification of serial ten-fold dilutions of a linearized plasmid containing the *ema-1* gene of *T. equi* in the presence (gray) or absence (black) of background DNA from uninfected equine blood. Individual Cq values were plotted against the *ema-1* gene copy number. In **d**, Cq values were plotted against the dilution factor of standards represented by DNA extracted from serial ten-fold dilutions of *B. caballi*-infected equine blood in uninfected equine blood (black) or DNA extracted from the undiluted sample of infected blood serially diluted in TE buffer (gray). Graphs were produced using Prism 6. *Abbreviations*: *R*^2^, coefficient of determination; E, amplification efficiency

Acceptable amplification efficiency was repeatedly achieved when serial dilutions of DNA extracted using the optimized protocol from stabilates of equine blood infected with either *T. equi* or *B. caballi* were prepared and amplified by the duplex qPCR on five separate days. Figure 2(a, b) shows standard curves generated from combined data of these five runs. The 95% CI of E values calculated for separate runs were 93.5–95.6% for *B. caballi* and 91.8–96.0% for *T. equi*.

Analytical sensitivity of the duplex qPCR assay for *T. equi* was assessed by amplification of serial ten-fold dilutions of linearized plasmid DNA containing from one million copies of the *ema-1* gene down to one copy. These standards were amplified in either the presence or absence of background DNA from uninfected equine blood. Individual Cq values of the runs were plotted against copy numbers of the *ema-1* gene (Fig. 2c). There was a statistically significant difference (*F*_(1, 47)_ = 5.169, *P* = 0.0276) between the slope values when calibration curves of standards amplified in the presence or absence of background DNA were compared. The E-value was lower in the presence of background DNA, but it still remained within the acceptance range. Every replicate of the dilution corresponding to 10 copies of the *ema-1* gene amplified even in the presence of background DNA with a mean Cq of 36.78 (95% CI: 36.09–37.46).

To assess the analytical sensitivity for *B. caballi*, the two sets of differently prepared infected blood-derived standards were amplified by the duplex qPCR and individual Cq values were plotted against template dilutions (Fig. 2d). There was no statistically significant difference between the slopes of these standard curves (*F*_(1, 44)_ = 0.4082, *P* = 0.5262), and the amplification efficiencies were all within the range of acceptance. All replicates amplified in the 10^-5^ dilution with mean Cq of 37.7 (95% CI: 35.69–39.71), whereas there was no amplification in the subsequent dilution. Therefore, given the starting 0.4% parasitemia in the blood used for this determination, the analytical sensitivity of the duplex qPCR assay for *B. caballi* was 4 × 10^-6^% infected cells.

### Evaluation of diagnostic performance

For the samples from the 362 presumably EP- negative horses from non-enzootic areas in Canada and USA tested by cELISA and the duplex qPCR, 100% diagnostic specificity was achieved for both assays, with all samples test-negative for both *T. equi* and *B. caballi*.

The relative diagnostic sensitivity and specificity of the duplex qPCR were assessed using samples collected from 430 horses of unknown infection status from an enzootic area for EP in Brazil. There was a fairly good agreement between *T. equi* infection prevalence values obtained using the molecular assays (duplex qPCR: 87.9%; *T. equi* 18S rRNA qPCR: 90.5%) and cELISA (87.4%) (Table 2). In contrast, there was a marked difference between *B. caballi* infection prevalence estimates determined using the qPCR assays (duplex qPCR: 9.3%; *B. caballi* 18S rRNA qPCR: 7.9%) and cELISA (58.6%). By cELISA, 230 of the 430 (53.5%; 95% CI: 48.8–58.1%) horses tested were demonstrated to have antibodies to both piroplasm species. As well, of the 40 horses positive for *B. caballi* by the duplex qPCR, 38 (95%; 95% CI: 82.6–99.5%) had *T. equi* (mixed) infections confirmed by both the duplex qPCR and single-target 18S rRNA qPCR for *T. equi*.

**Table 2:** Test results of the molecular assays and cELISA on samples from 430 Brazilian horses

Positive	*Theileria equi*			*Babesia caballi*		
	Duplex qPCR	18S rRNA qPCR^a^	EMA-1 cELISA	Duplex qPCR	18S rRNA qPCR^b^	RAP-1 cELISA
*n*	378	389	376	40	34	252
%	87.9	90.5	87.4	9.3	7.9	58.6
95% CI	84.5–90.7	87.3–92.3	84.0–90.2	6.9–12.4	5.7–10.9	53.9–63.2

^a^Kim et al. [30]

^b^Bhoora et al. [27]

**Fig. 3:**
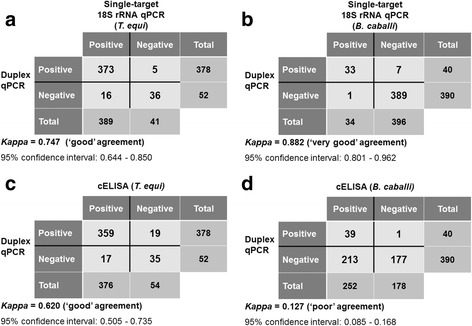
Level of agreement between test results of the duplex qPCR and those of the single-target 18S rRNA qPCR for *T. equi* (**a**), single-target 18S rRNA qPCR for *B. caballi* (**b**), cELISA for *T. equi* (**c**) and cELISA for *B. caballi* (**d**) performed on samples from 430 Brazilian horses

For a more comprehensive comparison of the test results, 2 × 2 contingency tables were created and the Kappa statistic was applied. By this analysis, a ‘good’ agreement was ascribed to the results of the duplex and single-target 18S rRNA qPCR tests for *T. equi* (Fig. 3a), with a *κ* value of 0.747. Discrepant results were obtained in 21 samples that showed high Cq values that were generally above 36 (mean Cq ± standard deviation (SD): 36.83 ± 1.67). There was a ‘very good’ agreement between test results of the duplex qPCR and the single-target 18S rRNA qPCR for *B. caballi* (*κ* = 0.882; Fig. 3b). Discordant results were detected in only 8 samples with Cq values above 36 (mean Cq ± SD: 38.0 ± 0.97). A ‘good’ agreement (*κ* = 0.620) was also achieved when the results of testing by the duplex qPCR were compared to those of cELISA for *T. equi* (Fig. 3c). Seventeen of the total 376 (4.5%) seropositive horses were identified as negative and 19 of 54 (35.2%) seronegative horses were identified as positive by the duplex qPCR. However, for *B. caballi*, the level of agreement between the duplex qPCR and cELISA test results was classified as ‘poor’ (*κ* = 0.127), as only 15.5% (39 of 252) of the seropositive horses tested positive by the molecular assay (Fig. 3d). Only one of the 178 antibody-negative animals was identified as positive for this piroplasm species by the duplex qPCR.

Assuming a prevalence of EP in Brazilian horses as determined by cELISA in this study, and given the values of relative diagnostic specificity and sensitivity of the duplex qPCR compared to cELISA as the reference method, positive predictive values of the molecular assay under conditions of high endemicity were nearly 100% for both *T. equi* and *B. caballi* infections, whereas negative predictive values for *T. equi* and *B. caballi* were 74.2 and 45.5%, respectively.

**Fig. 4:**
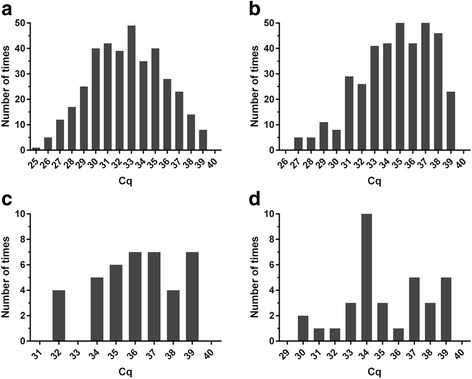
Frequency distribution of Cq values obtained by testing blood samples from 430 Brazilian horses for *T. equi* by the duplex qPCR (**a**) and the single-target *T. equi 18S rRNA* gene-specific qPCR (**b**), as well as for *B. caballi* by the duplex qPCR (**c**) and the single-target *B. caballi 18S rRNA* gene-specific qPCR (**d**). For samples with amplification plots produced in both duplicates, mean of these Cq values was used for the analysis, whereas when only one of the duplicates amplified, that single Cq value was used unaltered. Graphs were generated using Prism 6

The frequency distribution of Cq values obtained from testing of the samples from the 430 Brazilian horses by the molecular assays assessed in this study are shown in Fig. 4. There was a near-symmetrical distribution of Cq values of the duplex qPCR test results for *T. equi* with a peak at 33 (Fig. 4a). The lowest, median and the highest Cq values of this dataset were 25.5, 32.7 and 39.4, respectively. Respective Cq values of the single-target *T. equi* 18S rRNA qPCR dataset were slightly higher, namely 26.6, 35 and 39.5. The Cq value frequency distribution for this molecular assay reached a peak at 35 and was skewed towards higher Cq values (Fig. 4b). Although there was a very good agreement between test results when *B. caballi 18S rRNA* gene-specific primers and probe were used in the duplex or the single-target qPCR, analysis of the frequency distribution of Cq values suggests a slightly more robust performance of these oligonucleotides under the earlier published conditions (Fig. 4c, d) [27].

## Discussion

In this study, we determined the performance characteristics of a newly developed duplex qPCR assay for simultaneous detection of both causative agents of EP. This assay demonstrated 100% diagnostic specificity for both *T. equi* and *B. caballi* when blood samples from 362 seronegative horses and ponies located in non-enzootic areas for EP were tested. This study also showed that in 95.5% (95% CI: 92.8–97.3%) of horses from a highly enzootic area for EP in Brazil with *T. equi*-positive status, as confirmed by cELISA, parasitemia levels of this organism were at or above the detection limit of the duplex qPCR, supporting a high diagnostic sensitivity of this assay for *T. equi*. This agrees well with high diagnostic sensitivity estimates for molecular assays developed for other *Theileria* spp. (reviewed in [41]). Since parasitemia levels fluctuate over time, the diagnostic sensitivity of the duplex qPCR assay could be further improved by serial testing of initially cELISA-positive/qPCR-negative animals. Of the 54 Brazilian horses with no detectable antibodies to *T. equi* on cELISA, 19 (35.2%) tested positive for this piroplasm species in the duplex qPCR (Fig. 3c). This is consistent with other recently published data demonstrating significantly higher sensitivity of a PCR assay compared to cELISA in detecting early *T. equi* infections [12].

The *ema-1* gene of *T. equi* targeted by the duplex qPCR was originally described as a single-copy gene [42]. However, recently published genome sequence data for this piroplasm species revealed that *ema-1* belongs to a family of at least nine other genes [43]. Since these genes share comparatively low-level nucleotide identities with one another, it is likely that the *ema-1* gene-specific primers and hydrolysis probe target a single genomic locus. This would make these oligonucleotides especially suitable for quantitative assessment of *T. equi* loads by qPCR. The duplex qPCR assay reliably detected at least 10 copies of *ema-1* in the presence of background DNA from equine blood. This would roughly translate to one organism per microliter of blood, which is similar to analytical sensitivity estimates for other qPCR assays recognized as sensitive for the detection of equine or bovine piroplasm species [28, 30, 44].

Performance of the duplex qPCR assay was compared in this study to that of a previously developed single-target 18S rRNA qPCR for *T. equi* [30] using samples from Brazilian horses. The latter assay has been applied in numerous studies performed by various research groups, which substantiated its reliable performance [27, 45–47]. There was a good agreement in our study between the duplex qPCR and the 18S rRNA qPCR for *T. equi* (Table 2, Fig. 3a). The prevalence values obtained using these molecular assays (87.9 and 90.5%, respectively) agree well with the *T. equi* seroprevalence of 87.4% revealed in this horse population by cELISA. A higher proportion of horses identified as positive by the 18S rRNA qPCR may be due to a slightly higher analytical sensitivity of this assay compared to that of the duplex qPCR, as *T. equi* has two rRNA operons [43]. There might also be yet uncharacterized rare variants of the *ema-1* gene bearing polymorphisms in the hybridization sites for primers and/or probe developed in this study that interfere with assay performance. However, this might also apply to *T. equi*-specific qPCR methods targeting the *18S rRNA* gene, as comparatively high levels of sequence heterogeneity in this gene were revealed among different strains of *T. equi* [48–50].

By contrast, the level of concordance between the cELISA and duplex qPCR results from testing of Brazilian horses for *B. caballi* was poor (Table 2, Fig. 3d). However, this is not attributable to a less efficient performance of *B. caballi 18S rRNA* gene-specific primers and hydrolysis probe developed by Bhoora et al. [27] under the duplex qPCR conditions. The detection limit of the single-target 18S rRNA qPCR assay, defined as the level of *B. caballi* parasitemia at which 95% of positive samples were detected, has been determined to be 1.14 × 10^-4^% infected cells, corresponding to a Cq of 35.82 [27]. The performance of these oligonucleotides in the duplex qPCR was similarly robust, as evidenced by the following: (i) Cq values of ≤ 35.5 were obtained in all replicates of the dilution corresponding to 4 × 10^-5^% infected cells, and (ii) the results of testing field samples from a representative number of horses from Brazil, an enzootic area for EP, by both the duplex qPCR and the single-target 18S rRNA qPCR for *B. caballi* were in a very good agreement (Fig. 3b). A markedly higher level of agreement between this 18S rRNA qPCR assay and IFAT has been reported for testing of field samples from South African horses [27]. Of the 41 horses tested in that study, 29 (70.7%) had detectable antibodies to *B. caballi* and only three out of these seropositive horses tested negative by the 18S rRNA qPCR. In our study, only 34 out of 252 (13.5%) Brazilian horses with antibodies to *B. caballi* detectable in cELISA were identified as positive by the single-target 18S rRNA qPCR. The reason for such a noticeable difference in the proportion of seropositive horses identified as positive by this molecular assay for the two populations of horses tested remains unknown.

A significantly higher seroprevalence of *B. caballi* infection compared to that demonstrated by molecular assays has been reported in several other epidemiological studies [21, 29, 51–54]. In 487 horses from four states in Brazil, including the same region assessed in the current study, and tested for both causative agents of EP by IFAT and a duplex qPCR assay targeting the *ema-1* gene of *T. equi* and the *bc48* gene of *B. caballi*, high seroprevalence values of 91 and 83% were obtained for *T. equi* and *B. caballi*, respectively [29]. However, in both that and our current study, the number of horses that tested positive for *B. caballi* by molecular assay was only approximately one-sixth that of the animals with detectable antibodies to this piroplasm species. Significantly higher seroprevalences compared to prevalence estimates obtained using nPCR have also been reported for *B. bovis* and *B. bigemina* infections in water buffaloes from enzootic areas in Mexico and Egypt [55, 56].

In another study, a representative number of horses sampled in south-western Mongolia were tested for both *T. equi* and *B. caballi* using IFAT and a conventional duplex PCR [21]. By applying logistic regression models to the data, an age-dependent increase in prevalence as determined by PCR was shown for *T. equi* but a decrease was demonstrated for *B. caballi*. However, there was an age-dependent increase in the proportion of IFAT-positive horses for both piroplasm species. These data seem to support the contention that, unlike lifelong chronic infections caused by *T. equi*, horses might eventually clear infections with *B. caballi* without babesiacidal treatment. However, persistence of detectable levels of antibodies to *B. caballi* suggests the presence of viable parasites in the host, as supported by previous work that demonstrated that detectable antibodies specific to this piroplasm species wane within a few months after parasite clearance [34].

Interestingly, compared to the *B. caballi* prevalence estimates for Brazilian horses obtained in our study, there was a higher level of agreement between the numbers of seropositive and positive PCR results for Mongolian horses tested for *B caballi* [21], in spite of the conventional PCR that was used in that study, which generally has a lower sensitivity than qPCR. This may be attributable to several factors including differing dynamics of exposure to *B. caballi* for horses in these enzootic areas. More frequent exposure of horses to *B. caballi*-infected ticks is more likely to occur in Brazil where this pathogen is vectored by *Dermacentor nitens*, a one-host tick species that produces about three generations annually [57]. By contrast, immature stages of *D. nuttalli*, a known vector of this piroplasm species in Mongolia, generally feed on small mammals and infestations of horses with this tick species follow a seasonal pattern [58]. It remains to be determined whether a more frequent exposure to *B. caballi* confers better control over antigenically diverse parasite variants by the host’s immune system, resulting in reduced levels of parasitemia, analogous to the clinical course and pathogenesis of *Plasmodium* spp. infection in humans [59, 60].

*Babesia* species employ sequential modification of parasite-derived antigen expressed on the surface of parasitized erythrocytes as a strategy for resisting host immunity [61]. In some species of *Babesia* this surface-exposed antigen is also responsible for adhesion of infected red blood cells to vascular endothelium leading to sequestration of parasitized cells in the microvasculature of different host tissues. It is not known whether the reduction of parasitemia levels of *B. caballi* in persistently infected horses is influenced by this phenomenon.

The level of agreement between *T. equi* infection prevalence estimates for Brazilian horses determined by serology and qPCR in the study published by Heim et al. [29] was lower than that in our study. The higher diagnostic sensitivity of our duplex qPCR assay for *T. equi* may be attributed to a wider range of allele specificity of the *ema-1*-specific oligonucleotides developed in this study, and/or to the modifications that we introduced in our assay’s protocol to eliminate adverse effects of PCR inhibitors from blood. This study demonstrated that PCR inhibitors present in DNA extracted from equine blood using a widely-employed commercial protocol at levels that were not sufficient for complete abrogation of the amplification still caused a significant increase in Cq values (Fig. 1), thus affecting the assay’s sensitivity. This would also affect the accuracy of quantification by qPCR if serial dilutions of the standard used (e.g. purified plasmid DNA fragment or PCR product) were not subject to the same matrix effect. However, the problem can be mitigated by utilizing the modified DNA extraction procedure used here, or any other DNA extraction method that similarly entails a more thorough removal of inhibitory haem components from such DNA preparations.

## Conclusions

This study provides validation data for a new duplex qPCR assay for the diagnosis of EP that utilizes newly developed primers and TaqMan MGB probe for the *ema-1* gene of *T. equi*, and *B. caballi 18S rRNA* gene-specific primers and MGB probe adopted from an earlier study [27]. The advantage of this qPCR is that it is a multiplex assay with performance characteristics similar to those of well-established single-target qPCR assays recognized as sensitive for the detection of EP. The analytical sensitivity of this assay was confirmed to be high for both causative agents of EP, which should enable the detection of single or mixed infections with very low levels of parasitemia. The assay demonstrated high diagnostic specificity for both piroplasm species, as there were no false-positive reactions in testing of samples from a representative number of seronegative horses from non-enzootic areas for EP. Relative diagnostic sensitivity was similarly high (95.5%) for *T. equi* when a subset of Brazilian horses with antibodies to this piroplasm species detected by cELISA (*n* = 376) was analysed. However, only 15.5% of the 252 Brazilian horses with detectable antibodies to *B. caballi* had parasitemia levels at or above the detection limit of this molecular assay. Capillary blood sampling may need to be considered as a potential approach for improving the diagnostic sensitivity of molecular assays for *B. caballi*. Given savings in turnaround time and reagent costs provided by multiplexing in comparison to a single-target assay format, the duplex qPCR assay developed in this study is suitable for epidemiological surveys. However, the results presented here further demonstrate that prevalence studies performed using only molecular methods may significantly underestimate the prevalence of exposure to *B. caballi*. The data presented here support the suitability of the *ema-1*-specific oligonucleotides developed in this study for confirmatory testing of non-negative serological test results for *T. equi* by qPCR. However, for *B. caballi*, only positive molecular test results can be interpreted with confidence when determining the infection status of individual animals for disease control and regulatory purposes.

## Abbreviations

CFAP: Center for Food-borne and Animal Parasitology
EP: equine piroplasmosis
PCR: polymerase chain reaction
qPCR: quantitative real-time PCR
nPCR: ‘nested’ PCR
cELISA: competitive enzyme-linked immunosorbent assay
IFAT: indirect immunofluorescent antibody test
CFT: complement fixation test
EMA-1: equi merozoite antigen 1
RAP-1: rhoptry-associated protein 1
*bc48*: gene encoding a 48-kilodalton merozoite rhoptry protein of *B. caballi*
EDTA: ethylene diamine tetra-acetic acid
BSA: bovine serum albumin
MGB: minor groove binder
NFQ: non-fluorescent quencher
CI: confidence interval
Cq: quantification cycle
